# Metagenomic and Genomic Analyses Reveal Prevalent Spread and Evolution of the Bat White-Nose Pathogen *Pseudogymnoascus destructans* in Western Canada

**DOI:** 10.3390/jof12020154

**Published:** 2026-02-21

**Authors:** Yue Wang, Chadabhorn Insuk, Cory Olson, Jianping Xu

**Affiliations:** 1 Department of Biology, McMaster University, Hamilton, ON L8S 4K1, Canada; wangy660@mcmaster.ca (Y.W.); insukc@mcmaster.ca (C.I.); 2 Wildlife Conservation Society Canada, Edmonton, AB T5A 4Y6, Canada; colson@wcs.org

**Keywords:** *Pseudogymnoascus destructans*, metagenomics, genomics, single nucleotide polymorphisms, copy number variations, colony growth and morphology variations

## Abstract

Bats play a crucial role in the ecosystem. However, North American bat populations have experienced a dramatic decline since 2006 due to white-nose syndrome, a disease caused by *Pseudogymnoascus destructans* (*Pd*). This fungus can invade and damage the skin on bat wings and muzzles during hibernation. Since 2021, *Pd* has been reported at selected sites in western Canada, the region with the highest bat diversity in Canada, eliciting urgent calls for action among diverse stakeholders. Here we analyze nine metagenomes of bat guanos and wing swabs and the genomes of five *Pd* strains from western Canada to investigate the distribution and diversity of *Pd* in this region. *Pd* was found in all nine metagenomic samples and the metagenome sequences enabled us to identify the associated bat species. Divergence time estimates of *Pd* based on whole-genome sequences suggest that *Pd* likely entered Alberta two to five years before its first official report. Furthermore, we found evidence of abundant gene copy number variations in this species. Together, our metagenomic and genomic analyses indicate that *Pd* is more prevalent than currently recognized and is evolving and diversifying. Continued surveillance with more comprehensive methods is needed to accurately track its spread and facilitate timely management of white-nose syndrome in North America.

## 1. Introduction

Bats are a group of flying mammals of the order Chiroptera which exhibit great diversity with ~1500 recorded species worldwide [1]. Depending on the species, bats have a wide variety of dietary preferences, including fruit, nectar, and insects. Diverse diet choices make them a keystone species in many ecosystems around the globe, contributing to pollination, seed dispersal, and insect and infectious disease control. For example, the estimated value of pest control by bats in agricultural systems is approximately US $22.9 (3.7–53) billion per year in the United States alone [2]. However, populations of several North American bat species such as the little brown myotis (*Myotis lucifugus*), Indiana myotis (*Myotis sodalis*), northern myotis (*Myotis septentrionalis*), and tricolored bat (*Perimyotis subflavus*) have declined drastically due to white-nose syndrome (WNS), a deadly disease caused by the invasive ascomycete fungus *Pseudogymnoascus destructans* (*Pd*). Understanding the distribution and route(s) of *Pd* spread is essential for disease control and bat conservation.

Since the initial observation in 2006 in the state of New York in the US, WNS has quickly spread through populations of several North American bats [3]. For example, according to a study analyzing bat counts in North America between 1995 and 2018, *M. septentrionalis*, *M. lucifugus*, and *P. subflavus* declined by more than 90% in some geographic regions due to WNS [4]. Multilocus sequence typing and whole-genome sequencing revealed low to no genetic variation in *Pd* strains in North America, consistent with a recent single introduction followed by rapid clonal expansion across the continent [5,6,7]. In contrast, European isolates exhibited greater genetic diversity, and unlike those in North America, both mating types required for sexual reproduction are present in Europe. These results support the hypothesis that WNS was introduced to North America from Europe, likely through human-mediated transport.

One main contributor to the quick dispersal of such a disease is host species population connectivity. Many North American bat species are sociable and swarm in late summer and autumn for the purpose of mating and hibernation. This behavior facilitates gene flow but also increases the risk of disease transmission [8,9,10]. A study analyzing 637 *M. lucifugus* samples from 29 locations across North America identified a lower level of genetic differentiation across the regions where WNS had spread rapidly since its emergence [11]. This is further evidenced by the presence of a clonal *Pd* genotype throughout eastern and central Canada and the United States [6,12]. In western Canada, *Pd* was first found in Saskatchewan in 2021, followed by detections in British Columbia and Alberta in 2022 [13,14]. However, as of December 2025, WNS has not been reported in British Columbia, the westernmost province in Canada that also contains the highest bat species diversity. Together, the results highlight an urgent need to emphasize WNS monitoring, surveillance, and research on the evolution of *Pd* in western Canada.

Whole-genome sequencing (WGS) is a powerful technique to determine the complete genomic sequence of an organism. WGS contrasts with targeted sequencing methods which focus only on specific genes or genomic regions. In general, high-coverage WGS can provide a comprehensive view of an organism’s genome, but it entails higher costs and requires more computational power and bioinformatic expertise than targeted sequencing. Nonetheless, the information provided by WGS has played a critical role in identifying pathogens, monitoring epidemics, and revealing the diversity, adaptation, and population dynamics of pathogens [15,16,17,18]. Metagenomics is another sequencing technique that can analyze the entire genetic material from a sample [19]. This approach is more comprehensive than traditional culture-based sequencing of individual strains for identifying microbial diversities in samples, where only culturable species can be studied, and is more balanced than polymerase chain reaction (PCR)-based techniques such as DNA metabarcoding, where only DNA from specific genetic markers from a selected group of organisms can be analyzed [20,21]. The main limitations of metagenomic sequencing are the cost and bioinformatic expertise required. In addition, detecting rare taxa in a mixed sample also requires very high sequencing depth. Another method for pathogen detection is real-time quantitative PCR (qPCR) that can provide cost-effective, rapid, and sensitive detection in systems with well-characterized targeted genes. For the white-nose syndrome pathogen *Pd*, a qPCR method was established and has facilitated the analysis of a large number of samples and provided individual-level quantification of *Pd* loads, revealing the patterns of *Pd* spread over the last decade. However, false positives and false negatives can happen in qPCR. As a result, multiple repeats of qPCR for the same sample are often required, and critical threshold (Ct) values need to be established for consensus interpretation of qPCR results; however, some of these interpretative criteria are subjective. In contrast, metagenome sequencing generates clean readable DNA sequences that can be directly compared with reference samples for robust species identification and is robust to interpretation. Together, considering the strengths and limitations of both methods, a combination of metagenomic sequencing and qPCR may serve as complementary approaches with qPCR for large-scale surveillance and metagenome sequencing for robust identification and genetic analyses.

In this study, bat guano and wing swab samples from western Canada were analyzed using real-time qPCR and metagenome shotgun sequencing to screen for *Pd*. We hypothesize that metagenome sequencing can complement the current gold standard method real-time qPCR for detecting *Pd* in bat samples. In addition, we hypothesize that metagenome analyses of guano/wing swab could allow us to identify the bat species at the sites. Furthermore, we aim to obtain pure cultures of *Pd* from Alberta and their whole-genome sequences. We hypothesize that whole-genome sequences will identify mutations and copy number variations to allow us to explore the potential dispersal and genome evolution of *Pd* strains in western Canada and North America.

## 2. Materials and Methods

### 2.1. Metagenomic Sample Collection and Processing

This study analyzes bat fecal samples and wing swabs for the fungal pathogen *Pd*. The samples were collected as part of surveillance in Alberta, British Columbia, and Saskatchewan by the Wildlife Conservation Society Canada and provincial governments. Three British Columbia guano samples for metagenome analyses were collected in 2022 at Grand Forks and provided to us by the BC Ministry of Water, Land and Resource Stewardship. Two guano samples from Alberta and three guano samples from Saskatchewan were collected by the Wildlife Conservation Society Canada or its partners in 2023. All guano samples were collected below bridges or bat house roosts without disturbance to bats, except for sample ABP23_LW001, which was collected from a bat-holding bag. One bat wing swab sample was collected in 2023 for metagenome analysis and in 2024 for the isolation of pure *Pd* cultures by the Wildlife Conservation Society Canada, in collaboration with Alberta Environment and Protected Areas. All wing swab samples were collected following the standard protocol as outlined in [22], under Alberta Research Permit Numbers 23-204 and 24-285. Bats were captured near known roosting areas using mist nets of varying lengths and configurations, in accordance with applicable animal care protocols for the province [23].

Collected samples were directly sent for real-time qPCR to the Animal Health Laboratory at the University of Guelph [24]. Biological samples were considered positive for *Pd* if the cycle threshold (Ct) ≤ 35, inconclusive if 35 < Ct ≤ 40, or negative if Ct > 40 [24].

Among the nine guano/wing swab samples, ABPD23_LW001 and ABPD23_0708 were pooled from two original samples each from the same site and time with similar real-time qPCR Ct values to ensure sufficient DNA for metagenomic sequencing. All samples were sequenced at Metagenom Bio Life Science Inc. (Waterloo, ON, Canada) using the Illumina Hiseq platform.

### 2.2. Pure-Cultured Strain Collection and Processing

For obtaining pure *Pd* strains from Alberta, 21 bat wing swabs (suspended in 450 µL Tris-EDTA buffer, pH 8.0) and 22 bat guano samples (5 pellets in 450 µL Tris-EDTA buffer, pH 8.0, homogenized by high-speed vortexing) were inoculated (100 µL) and cultured on Sabouraud dextrose agar (SDA) supplemented with 30 mg/mL chloramphenicol and 30 mg/L streptomycin. The swabs used for culturing were separate from those used for metagenome sequencing. Specifically, spread plating was done by adding 100 µL of wing swab or guano suspended in sterile distilled water onto the agar surface and incubated at 15 °C for 14–30 days. The colonies were purified on (SDA) until pure *Pd* cultures were obtained. *Pd* cultures were confirmed by the qPCR method, following the method described previously [24]. As shown below, we could only obtain *Pd* colonies from wing swab samples but not guano samples.

For whole-genome sequencing of the pure *Pd* strains, their genomic DNA was extracted following the cetyltrimethylammonium bromide (CTAB) DNA extraction method [25]. Briefly, this method involves treating mycelial mats from cultured *Pd* with liquid nitrogen and grinding the mycelia using a micropestle. CTAB lysis buffer with β-mercaptoethanol was added to the crushed samples and samples were incubated at 65 °C. Subsequently, organic extraction of DNA was done using chloroform: isoamyl alcohol (24:1) and samples were centrifuged to recover the aqueous phase. The DNA was then precipitated with ice-cold absolute isopropanol, washed with 70% ethanol, and finally resuspended in 50 uL Tris-EDTA buffer (pH 8.0). Extracted DNA was quantified using a Qubit 4 fluorometer (Thermo Fisher Scientific, Mississauga, ON, Canada) to assess DNA concentration. DNA integrity and purity was verified by agarose gel electrophoresis (1% agarose, 120 V). The whole-genome sequencing libraries were prepared using the Illumina DNA Prep Kit (Illumina, San Diego, CA, USA) following the manufacturer’s instructions, including DNA fragmentation, end-repair, adapter ligation, and library amplification steps [26]. Libraries were sequenced on an Illumina NovaSeq 6000 platform to generate 150 bp paired-end reads at Metagenom, Inc (Waterloo, ON, Canada).

### 2.3. Sequence Analyses

Genomic analyses were conducted on the nine biological samples, five Alberta *Pd* strains, and the archived global samples that we retrieved from GenBank. For the nine biological samples, we first extracted the *Pd* reads by aligning the sequences to the reference genome ASM164126v1 using bowtie v2 [27]. To reduce false positives, the program was set to end-to-end mode, and the score-min was set to L, −0.06, −0.06. Then, the mapped reads were converted to the fastq format using SAMtools v1.13 [28].

The extracted *Pd* sequence reads from metagenomic data and the WGS data from pure-cultured strains were analyzed as follows. First, adapters and low-quality reads were removed using Trimmomatic v0.39 [29]. Clean reads were mapped to the reference genome (ASM164126v1; genome size: 35.8 Mb) using BWA-mem v0.7.17 [30] and the alignments were formatted using SAMtools v1.13. Variants were identified using GATK v4.2.5 [31] HaplotypeCaller in GVCF mode and merged using CombineGVCFs for all samples. Variants were then genotyped using GenotypeGVCFs. Single-nucleotide polymorphisms (SNPs) were extracted and filtered using SelectVariants and VariantFiltration respectively. The filtrations include quality relative to depth (QD < 2.0), strand bias (FS > 60.0, SOR > 3.0), mapping quality (MQ < 40.0, MQRankSum < −12.5), and positional bias of alternate reads (ReadPosRankSum < −8.0).

### 2.4. Bat Species Identification

In this study, we were interested in whether the metagenome sequence contained the host bat DNA that could be used to trace its bat species of origin. COI is an ideal DNA marker for species identification due to its presence in the highly copied mitochondrial genome and high interspecies variation. This is especially useful for metagenomic data, where DNA is often degraded or mixed from multiple species. There are 18 recorded bat species in Canada (https://batwatch.ca/sp_canada; accessed 10 January 2026). Their COI reference sequences were retrieved from the Barcode of Life Database (BOLD) system [32]. In the Supplementary Materials, Table S3 lists the geographic distribution and corresponding COI reference sequences of the 18 bat species [31]. Metagenomic reads were preprocessed in the same way as mentioned above for *Pd*; however, the reference sequences were replaced with COI sequences of the 18 Canadian bat species. For each sample, the source bat species was determined if its reads mapped to the entire reference sequence with a substantial average read depth and a very low variant frequency, less than 1% of the reference sequence.

### 2.5. Phylogenetic Analyses

Three phylogenies were generated to reveal the genetic relationships of Canadian *Pd* strains and to compare them with global strains. Maximum likelihood phylogenetic trees were constructed using IQTree v2.0.7 [33] with the GTR+G model and 1000 bootstrap replicates based on SNP alignments. SNP alignments were generated using Vcf2phylip v2.0 [34], with only loci containing unambiguous nucleotide calls in at least 10% of the individual samples retained.

### 2.6. Divergence Time Estimation

Divergence times of Alberta *Pd* strains from other strains were estimated using BEAST v2.7.8 [35]. Collection data were incorporated to calibrate the molecular clock. The site model was determined via BEAST model testing, and the substitution model included estimates of the mutation rate and transition/transversion ratio. A strict molecular clock was chosen with the substitution rate priorly set to 1 × 10^−5^, and a coalescent exponential population model was used to account for population dynamics. MCMC simulations were run for 20,000,000 steps, with parameters sampled every 1000 steps, and the first 10% of steps were discarded as burn-in. Tracer was used to assess convergence of the MCMC chain. Posterior distributions of divergence times were summarized using the TreeAnnotator in BEAST v2.7.8 [35], and the resulting maximum clade credibility tree was visualized in FigTree v1.4.4 (http://tree.bio.ed.ac.uk/software/figtree/, accessed 1 December 2025).

### 2.7. Copy Number Variation Analyses

The gene copy number was estimated for all *Pd* strains with WGS data. The reference genome comprises 9620 annotated genes. Gene symbols and coordinates were retrieved from the GFF file (https://www.ncbi.nlm.nih.gov/datasets/genome/GCF_001641265.1/; accessed 10 November 2025). The average read depth of each gene and scaffold was measured using SAMtools. The gene copy number was estimated as the ratio of the gene’s average read depth to the scaffold’s average read depth. The coefficient of variation was used to select genes that have high copy number variation across the samples. Only genes with at least 1000 bp were considered.

### 2.8. Four-Gamete Test for Recombination Detection

To assess evidence of potential recombination among *Pd* strains, the four-gamete test was applied to biallelic SNP loci using methods described previously [36]. For each pair of SNP sites, the presence of all four possible allele combinations (AB, Ab, aB, ab) was examined. A SNP pair exhibiting all four gamete genotypes was interpreted as evidence of at least one potential historical recombination event between the loci, while SNP pairs with fewer than four gamete genotypes were considered consistent with clonal reproduction and inconsistent with recombination. The proportion of SNP pairs showing four gamete genotypes was calculated for individual sample sets to estimate the extent of possible recombination in each population.

### 2.9. Phenotype Determination

Previous studies revealed several colony growth phenotypes were associated with *Pd* adaptation along geographic gradients [6,37]. To determine these colony growth phenotypes for the five new *Pd* isolates from Alberta, we first grew them on an SDA plate at 15 °C for three weeks until the *Pd* cultures sporulated. *Pd* spores were then harvested, and their concentrations adjusted to 10^6^ spores/mL. Then, 10 µL of the spore suspension was inoculated into the center of a fresh SDA plate and incubated at 15 °C for four weeks. The inoculated plates were set in triplicates for each *Pd* isolate. We characterized the phenotypes of *Pd*, including colony size (mm), pigment diffusion through agar (mm), colony surface exudate production (µL), exudate pigmentation, and colony surface mycelial pigmentation using the method described in [37]. In brief, colony sizes were estimated by using the spot densitometry function in FluorChem 8900 (Alpha Innotech, San Leandro, CA, USA). Similarly, pigmentation on both the colony surface and its diffusion in agar was approximated using the spot densitometry function. Exudate pigmentation was observed visually, and colony surface exudate production was quantified by micropipetting.

## 3. Results

### 3.1. Sample Information

We completed metagenomic analysis for nine bat guano and wing swab samples from western Canada, with three samples each from Alberta, British Columbia and Saskatchewan. Because of low DNA content, two of the nine samples were generated by pooling pairs of samples from the same location and date that had originally been collected and tested separately: one was a combined guano and wing swab sample from a bat in a holding bag (ABPD23_LW001) and another was a guano sample (ABPD23_0708). The original samples used to form pooled samples had identical critical threshold values for *Pd* in qPCR analyses. In addition, five *Pd* strains were isolated from bat wing swabs collected in Alberta in 2024 and their whole-genome sequences were obtained using the Illumina HiSeq platform. Strain and sample details are listed in Supplementary Materials, Table S1. These generated sequences were analyzed and compared with *Pd*-genome sequences reported previously from North America and other parts of the world. NCBI accession numbers and details of retrieved global strains used for comparisons are displayed in Supplementary Materials, Table S2.

### 3.2. Source Bat Species

For the nine guano/wing swab samples, we used DNA sequences of the mitochondrial cytochrome C subunit I (COI) gene within the metagenome sequences to identify the bat host species. Specifically, COI reference sequences from all 18 bat species occurring in Canada were retrieved from GenBank and confirmed through the Barcode of Life Database (BOLD), and these sequences were used to compare with those in the metagenome samples (Supplementary Materials, Table S3). In the Supplementary Materials, Table S4 lists the mapping results of metagenomic sample reads to the COI reference sequences from each of the 18 Canadian bat species. The comparisons identified three bat species associated with the nine guano/wing swab samples from western Canada, including *Myotis lucifugus, Myotis evotis*, and *Eptesicus fuscus*. Each sample has 1–2 COI reference(s) fully covered with the metagenome reads, with read depth ranging from 46.3 to 1817.2 and SNP ratio between 0.15%–0.76%. *Myotis lucifugus* was identified from eight of the nine samples except for BC22_3024_Pd_minus guano, for which *Myotis evotis* was detected. While sample SKPD23_180, collected from under a bridge and not a bat-holding bag, was found to be associated with both *M. lucifugus* and *E. fuscus*. Together, these results show that metagenome sequencing of guano and wing swab samples can be used effectively to identify the host bat species, including multiple bat species in a mixed guano sample.

### 3.3. Pd Identification from Bat Biological Samples

**Figure 1:**
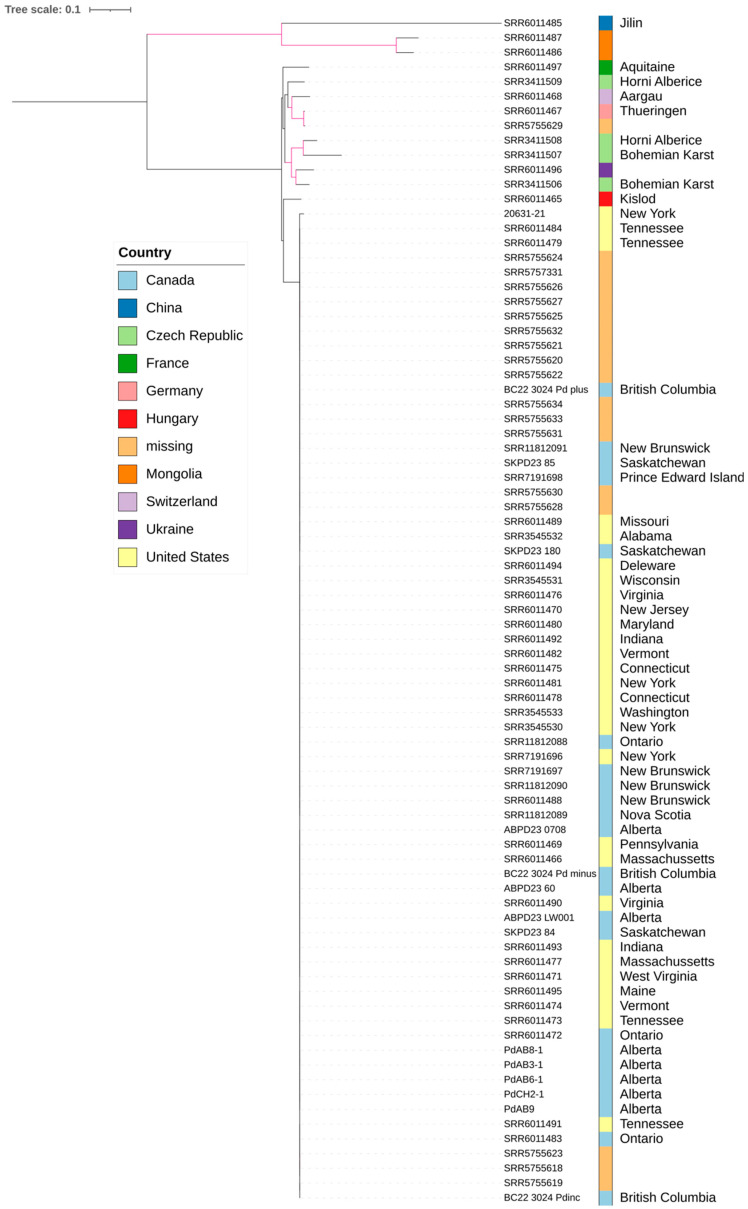
Phylogenetic tree of 80 *Pd* samples and reference strain 20631-21 based on 217,520 SNP loci with each SNP present in at least 10% of all samples. The 80 *Pd* samples comprise 9 metagenomic samples from western Canada, 13 non-North American strains, and 58 North American cultured strains. Geographic information is labeled as color strips. Branches highlighted in pink have at least 90% bootstrap support. Genbank accession numbers were used to represent previously published strains and when available, the corresponding geographic locations are given. Different colors represent different countries while the names to the right of colored bars represent state/province within each country. For new data reported in this study, sample or strain names corresponding to those in Table 1 and Table 2 are presented. Labels of the five pure-cultured Alberta strains are formatted as PdAB or PdCH with numbers, whereas the nine metagenomic samples are labeled according to their sampling locations (i.e., BC, AB, or SK). The scale bar represents the number of substitutions per site inferred from the SNP alignment.

To assess whether *Pd* can be identified from bat guano/wing swab samples through metagenome sequencing, we mapped metagenomic sequence reads to the *Pd* reference genome. This analysis revealed *Pd* sequences in all nine guano/wing swab samples, including the sample that tested negative for *Pd* in real-time qPCR. Among the nine samples, the *Pd* read-covered portion of reference genomes ranges from 25,459 bp to 780,642 bp with an average read depth spanning from 2 to 23 (Table 1). Given a reference genome size of 35.8 Mb, each sample has sequence reads mapped to only ~0.07–2.18% of the genome. The incomplete coverage and low read depth in some genomic regions are expected due to the high organism complexity and uneven read distribution among species within each sample. A maximum likelihood phylogeny was constructed by including *Pd* sequences in the nine metagenomic samples from western Canada, whole-genome sequences of the five pure-cultured *Pd* strains from Alberta, and the whole-genome sequences of 66 global strains retrieved from GenBank (Figure 1). Based on the available genomic regions, *Pd* sequences from bat guano/wing swabs in western Canada appeared to cluster unambiguously with the North American strains (Figure 1).

### 3.4. Divergence Time Estimates

**Figure 2:**
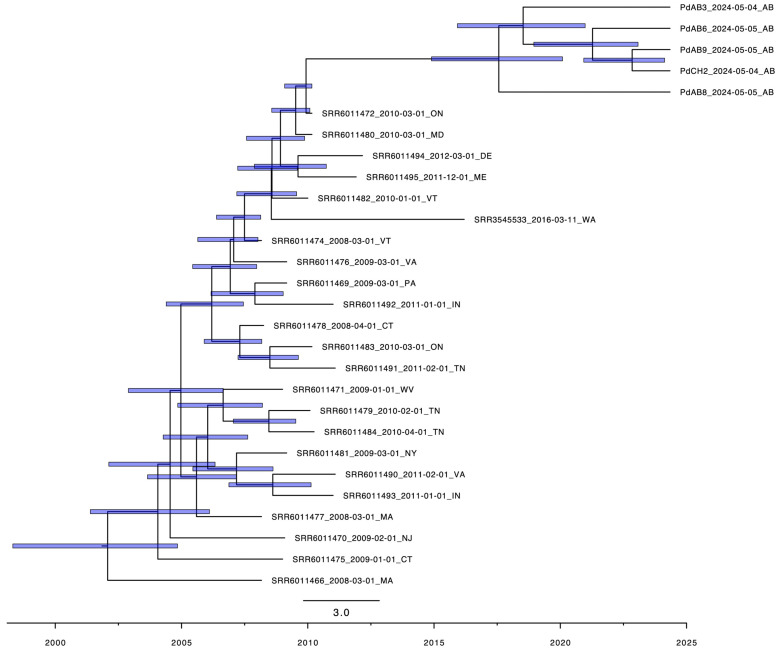
Maximum clade credibility tree for isolates of Pd from North America based on 2328 SNP sites showing divergence time estimates under a strict molecular clock model (GTR+G). Node bar represents 95% high probability density interval. Branch lengths are scaled to time, with node heights representing common ancestor heights. Strain labels are formatted as: strain ID_date of collection_abbreviation of the sampling location. AB: Alberta; ON: Ontario; DE: Delaware; MD: Maryland; VT: Vermont; ME: Maine; VA: Virginia; PA: Pennsylvania; IN: Indiana; CT: Connecticut; TN: Tennessee; NJ: New Jersey; WV: West Virginia; NY: New York; MA: Massachusetts; WA: Washington.

We estimated the divergence time between western Canadian *Pd* strains and those from eastern Canada and the US. Due to the incomplete *Pd* sequences and low-read depths of the *Pd*-genome-covered regions in the nine metagenome samples, only the whole-genome sequences of the five Albertan pure-cultured strains were used for the divergence time estimation. For this estimate, we first built a maximum clade credibility tree based on 2316 SNP sites that included the five pure-cultured Alberta and 23 representative North American strains with high-quality genome sequence data and with detailed time and location information from where the samples for these isolates were collected (Figure 2). After 20,000,000 Markov Chain Monte Carlo (MCMC) simulations, all parameters had effective sample sizes of >1000, indicating adequate mixing and convergence of the MCMC chains. Maximum clade credibility was summarized after discarding the 10% burn-in. Our analyses showed that the five Albertan *Pd* strains shared a recent common ancestry that had diverged from those in eastern North America and the state of Washington, with an estimated root age dated to approximately July 2017 (95% highest posterior density range: 2015–2020). This result indicated that *Pd* strains were likely present in Alberta at least two years before it was first detected in a 2022 sample by qPCR and reported in 2023.

**Table 2:** Pairwise genome-wide single nucleotide differences among five Alberta *Pd* strains.

Strain	PdAB3-1	PdAB6-1	PdAB8-1	PdAB9	PdCH2-1
PdAB3-1	0	126	110	153	148
PdAB6-1		0	130	134	133
PdAB8-1			0	111	126
PdAB9				0	103
PdCH2-1					0

### 3.5. Genomic Analyses of Pure-Cultured Strains

For the five pure-cultured *Pd* strains from Alberta, genomic SNPs were identified and compared with 66 global strains. When compared to the reference genome, a total of 217,757 SNP loci were identified among the global *Pd* samples. The pairwise SNP differences between strains range from 103 to 152,009. The top three strains that are most divergent from the North American strains are SRR6011485, SRR6011487, and SRR6011486, isolated from China and Mongolia. They differ from the other strains by 124,466–152,009 SNPs (Supplementary Materials, Table S5). SRR6011486 and SRR6011487, both from Mongolia, are more closely related to each other (38,224 SNPs) than to the Chinese strain SRR6011485 (116,450 and 119,370 SNPs respectively). As expected, the five Albertan strains exhibit very close relationships to each other, with the fewest SNP differences, ranging from 103 to 153 (Table 2). They differ from other strains by 299–135,455 SNPs (Supplementary Materials, Table S5).

**Figure 3:**
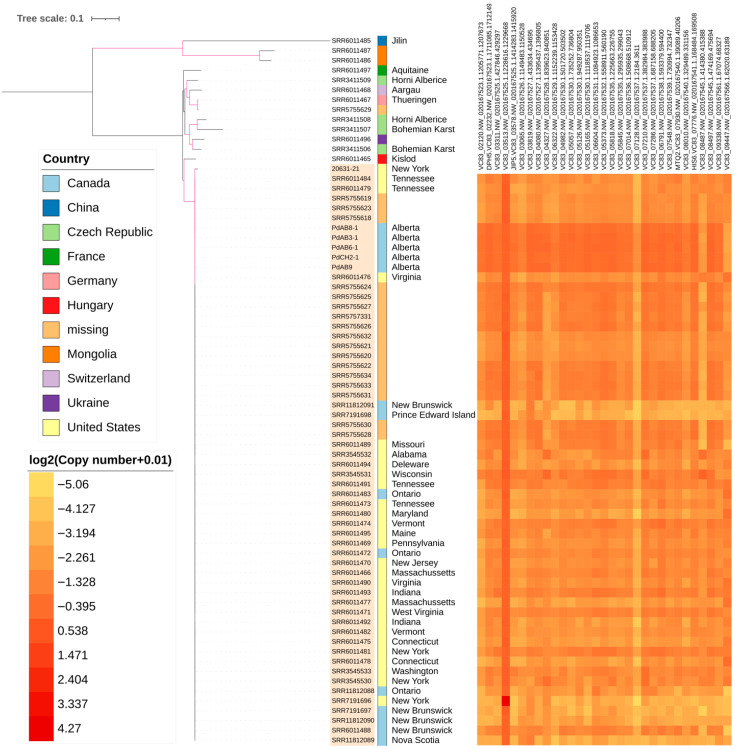
Phylogenetic tree of 71 samples and reference strain of *Pd* (20631-21) based on 217,500 SNP loci present in at least 10% of all samples. Geographic information is labeled as color strips. Heatmap representing copy number variation of 31 genes exhibiting high variability (coefficient of variation ≥ 0.5) among the North American dataset (Supplementary Materials, Table S6). Branches highlighted in pink have at least 90% bootstrap support. A genetic cluster with closely related strains from North America is highlighted in orange. The labels of the five pure-cultured Alberta strains are formatted as PdAB or PdCH with numbers, while Genbank accession numbers starting with SRR are used to represent previously published strains. Tree scale indicates nucleotide substitution per polymorphic site.

Aside from SNPs, we assessed the copy number of all genes present in the reference genome based on relative read depth within the WGS data. Within the reference genome, there are 9620 annotated genes (Supplementary Materials, Table S6). To improve the reliability of copy number detection, we applied a 1000 bp cutoff to reduce noise from short genes, low coverage, and mapping errors, resulting in 6717 genes retained. In the global *Pd* sample, 804 genes showed substantial copy number variability, with a coefficient of variation ≥0.3. When only North American strains were considered, 361 genes showed large differences in relative read depths. Among these 361 genes, 31 had a coefficient of variation ≥0.5 and the estimated copy number distribution across strains in these 31 genes is shown on a heatmap (Figure 3, Supplementary Materials, Table S7). Detailed functional annotations of these 31 genes are shown in Supplementary Materials, Table S7. The majority of these 31 genes are predicted as involved in DNA binding, protein function and protein metabolism. As expected, while differences were consistently observed between the Albertan strains and those from outside of Alberta, there was limited variability in gene copy number among the five pure-cultured Alberta strains (coefficients of variation all <0.15).

Maximum likelihood analysis indicates strong bootstrap support for a cluster containing strains from North America only (Figure 3). European strains have longer branch lengths relative to one another than those among North American strains, indicating greater genetic diversity and a longer evolutionary history in Europe (and Eurasia in general). Among the European strains, that from Hungary showed the closest genetic relationship with North American strains, likely representing part of an ancestral population that gave rise to North American *Pd* strains. Additionally, strains from China and Mongolia are the most divergent ones from the rest, forming the basal lineage to strains from Europe and North America.

### 3.6. Divergence, Dispersal, and Limited Recombination of Pd Within North America

**Figure 4:**
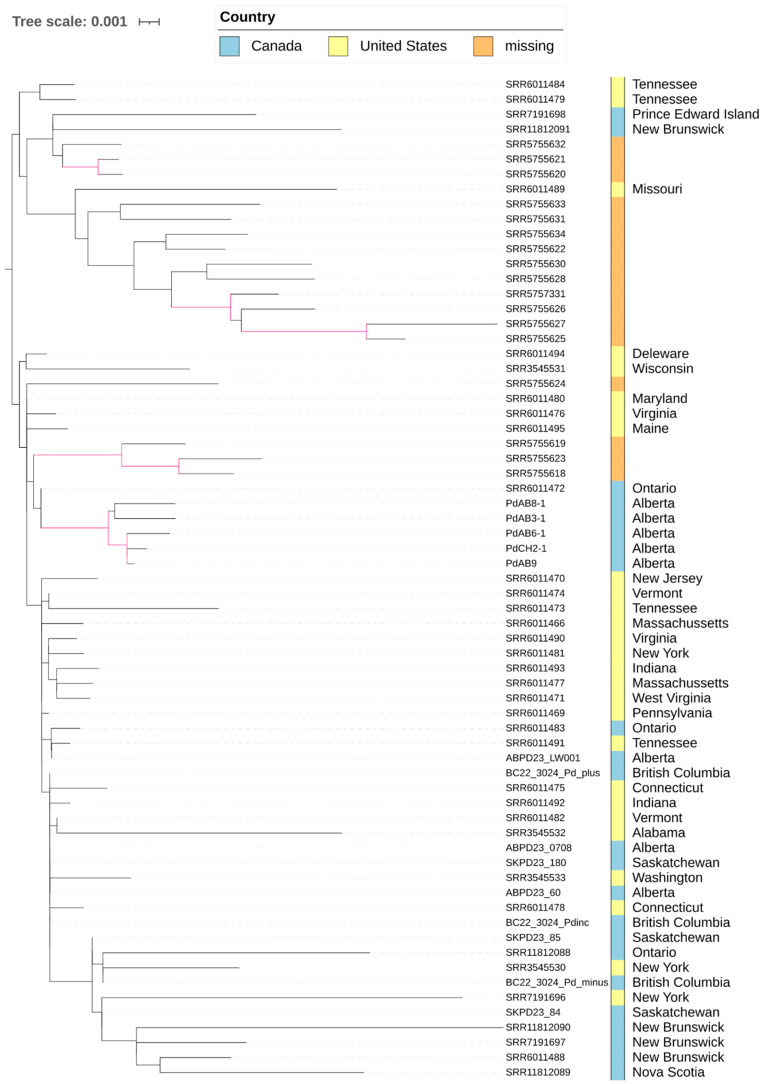
Phylogenomic relationships among nine metagenome samples of *Pd* and 58 *Pd* strains representing the North American cluster in Figure 3. The phylogeny is based on 3002 SNP loci present in at least 10% of the 67 samples. Branches highlighted in pink have at least 65% bootstrap support.

To understand the relationships among North American strains and infer the potential patterns of dispersal, we obtained a high-resolution phylogeny of North American strains by limiting the sample set to just the North American cluster and based on only SNP loci present in this sample set (Figure 4). The phylogeny indicated both geographic clustering and evidence for long-distance dispersals. Similar to the results shown above, the five Alberta strains formed a tight cluster with 65% bootstrap support, suggesting these strains likely originated from a recent common ancestor. However, these sequences also indicate that the western Canadian *Pd* metagenome sequences might be widely dispersed among various clonal lines within the North American *Pd* population (Figure 4). Of note, due to partial genome recovery and uneven read depth across samples from metagenomic data, Figure 4 only suggests broad genetic relationships and potential dispersal patterns of *Pd* strains from guano/bat wing samples in western Canada.

Interestingly, of the 744 biallelic SNP loci identified across the 58 North American strains, 0.437% SNP pairs show four combinations of alleles, consistent with parallel mutation and/or limited recombination among North American strains (Table 3). In contrast, the global sample contains 3.578% SNP pairs consistent with recombination and/or parallel mutation, which is more than eight times higher than in the North American population. No evidence of recombination was detected among the five Albertan strains.

### 3.7. Colony Growth Phenotypes

The fungal colony growth phenotypes were analyzed for the five Albertan *Pd* strains. Our analyses revealed significant differences in all three quantifiable phenotypic characteristics between at least one of the strain pairs (Table 4). For example, strains PdAB3, PdAB6, and PdAB8 produced larger amounts of diffusible pigments than strains PdAB9 and PdCH2. Additionally, PdCH2 produced the greatest exudate volume and the least pigmentation.

## 4. Discussion

In this study, we analyze bat guano and wing swab samples to detect *Pd* and investigate *Pd* evolution in western Canada. In total, 14 samples were analyzed, including nine guano/wing swab samples and five pure-cultured strains from bat wing swabs. Our metagenomic and genomic analyses of these samples reveal broader and earlier distribution of *Pd* in western Canada than officially reported. In addition, global *Pd* genomes seem highly variable, with evidence for large differences in gene copy numbers across its geographic range, including the rapid copy number changes since its arrival in North America.

Based on metagenome sequence, the nine guano/wing swab samples were found associated with *M. lucifugus*, *M. evotis*, and *E. fuscus*. Five pure Albertan *Pd* strains were isolated from *Pd*-infected wings of *M. evotis* and *M. lucifugus*. According to Neighbourhood Bat Watch (https://batwatch.ca/sp_canada; accessed 10 January 2026), all three bat species occur in Canada and have been observed in Alberta, British Columbia, and Saskatchewan. Among them, *M. lucifugus* has the broadest geographic distribution and has been observed in all Canadian provinces and territories. Our results indicate that bat species can be effectively identified from metagenome sequences of bat guano and wing swabs, similar to those based on COI sequences using targeted PCR and DNA sequencing of bat wing punch samples.

In North America, *Pd* was first reported in New York in 2006. In-depth investigations revealed a high incidence of cutaneous fungal infection in 105 out of 117 necropsied bats, which included 91 (out of 97) *M. lucifugus*, 8 (of 9) *M. septentrionalis*, 0 (of 5) *E. fuscus*, 3 (of 3) *Perimyotis subflavus,* and 3 (of 3) unidentified species [38]. Shortly after, this fungus was reported in Ontario in 2010 [39]. In western Canada, *Pd* has been reported in all three provinces since 2021 [14]. While these represent only the cases that have been sampled and detected, there may be many more undetected. In our study, qPCR analyses following the standard protocols for *Pd* detection confirmed *Pd* in four of the nine guano/wing swab samples. In contrast, metagenomic sequencing successfully captured and sequenced *Pd* DNA from all nine samples. Despite this, we must admit that the accuracy is attributed to the deep sequencing depth which can significantly increase the cost of this approach. The number of nucleotide bases sequenced for the nine metagenomic samples ranged from 17.88 to 61.95 Gb and their total cost was about $20,000 Canadian. In addition, metagenomic data introduce substantial analytical complexity due to their high dimensionality and taxonomic diversity. Thus, metagenomic sequencing can be considered a complementary tool to qPCR, particularly valuable for early detection, retrospective analyses, and comprehensive ecological assessments. Together, these findings suggest that *Pd* is more prevalent than previously thought. We note that there was no correlation between *Pd*-mapped metagenomic read counts and qPCR amplification cycles among the nine samples, which showed discrepancies between the two methods. Specifically, samples with high numbers of *Pd* DNA sequence reads in the metagenomes did not have low Ct cycles in the qPCR. Such a result is understandable because a sample containing abundant *Pd* DNA may also contain very high concentrations of DNA from other organisms, resulting in a relatively low number of *Pd* reads in the metagenome sequence file when each sample was sequenced to a similar read count.

Molecular dating analysis using the Albertan and other North American strains (Figure 2) indicates that *Pd* could have spread to western Canada by 2015, over six years before the first official report in that region. Our results call for updated guidelines to strengthen the monitoring and surveillance of *Pd* and WNS in western Canada.

We investigated the genetic relatedness among *Pd* strains by analyzing WGS SNP data. The 71-strain phylogeny revealed varying levels of genomic divergence among the strains, from highly divergent to closely related. In agreement with a previous study [5], Asian strains (within-group SNP range: 38,224–119,370) exhibit the most genetic divergence followed by European (21,272–75,460) and North American strains (103–11,427). In addition, the gene copy number distribution is highly consistent among North American strains, consistent with recent clonal expansion in this region (Figure 3). There are 3.578% of SNP pairs in the total sample set exhibiting all four gametes while only 0.437% four-gamete SNP pairs are present in the North American *Pd* population. These results support the conclusion that North American *Pd* emerged from a recent common ancestor and experienced a clonal spread consistent with previous reports [6,7,12,40]. The findings may reflect occasional recombination events in the North American *Pd* population, but additional data would be required to substantiate this hypothesis. SNP differences between European and North American strains range from 26,710 to 76,118, similar to the within-group SNP differences among European strains. However, North American strains differ from Asian strains by 124,466 to 138,472 SNPs, in agreement with previous reports that the North American *Pd* population was introduced from Europe [5,41]. The five pure-cultured Alberta strains fall into a tight cluster within the North American *Pd* clone. Their tight clustering of the Albertan strains reflects their recent shared ancestry.

Copy number variations contribute to genomic diversity and may provide additional insight into population structure beyond SNP-based analyses. Additionally, they may underlie organism adaptation by modulating gene dosage, increasing genetic diversity, allowing rapid response to environmental pressures, and providing material for new functions. Among 6717 genes with a length of at least 1000 bp, we found that approximately 800 showed copy number variation (coefficient of variation ≥ 0.3) in the total sample set. Notably, 361 genes showed copy number variation among North American strains (coefficient of variation ≥ 0.3). We further examined the genes with a high coefficient of variation (≥0.5) in copy number variation among the North American dataset and retrieved their Gene Ontology annotations from the NCBI Gene database. While many of the copy-number variable genes are unannotated, the annotated and hypothetical functional genes were dominated by protein/DNA/RNA-binding functions, ubiquitin ligase and oxidoreductase activities, and methyltransferase activity (Supplementary Materials, Table S7). One annotated gene encodes S-adenosylmethionine-dependent methyltransferase (*Mtq2*). In Baker’s yeast *Saccharomyces cerevisiae,* a strain with *MTQ2* deletion mutation has been shown to exhibit high resistance to inorganic arsenic, which generates oxidative stress inside pathogens [42]. However, while our exploratory CNV analysis identifies a few putative genes related to adaptation, these findings require further validation to confirm their functional or adaptive roles.

The five Albertan *Pd* strains show variations in several colony phenotypes, such as diffusible pigment production, exudate production, and exudate pigmentation. When compared with the strains reported in 2018 [37], phenotypic variations among the five isolates in this study are similar to or greater than those observed in strains collected from diverse locations across eastern North America before 2016. In particular, while growth rates are comparable to those reported previously, variations in pigmentation and colony morphology are greater among the newly analyzed strains. These results suggest further adaptations of *Pd* in Alberta, and likely in other regions of North America.

In conclusion, our findings demonstrate that *Pd* is more prevalent in bat populations in western Canada and likely arrived there much earlier than officially reported. While qPCR remains the standard method for routine diagnostic testing of *Pd* in provincial laboratories due to its cost-effectiveness and accessibility, our findings demonstrate that metagenomic sequencing provides excellent sensitivity for detecting *Pd* in bat guano and wing-swab samples, including potentially strain genotype identification. Given its effectiveness in early detection of *Pd*, metagenomics should be implemented as a complementary tool in cases where early detection is a priority. In addition, metagenome sequencing of guano and wing swabs allowed us to identify the associated bat species. The results of our genomic analyses are consistent with previous results showing that the North American *Pd* population was recently introduced from Europe, but has been evolving rapidly, accumulating significant genomic and phenotypic variations. Our results call for enhanced surveillance and research on WNS and *Pd* in western and other parts of North America, where *Pd* has yet to be officially reported, to more effectively manage the threat posed by this fungal pathogen [43].

## Figures and Tables

**Table 1:** Real-time quantitative PCR Ct values and *Pd*-genome coverage metrics of metagenome sequences for the nine samples of bat guanos and wing swabs from western Canada.

Sample	Real-Time Quantitative PCR Ct Value	Size of *Pd*-Genome-Covered Region (bp)	Average Read Sequence Depth of *Pd*-Genome-Covered Region
ABPD23_0708	31 *	85,347	3.18422
ABPD23_60	37 **	36,093	10.196
ABPD23_LW001	39 **	780,642	2.18864
BC22_3024_Pd_inc	38 **	82,412	19.5281
BC22_3024_Pd_minus	ND ***	66,171	23.4417
BC22_3024_Pd_plus	32 *	101,866	18.9948
SKPD23_180	30 *	151,062	9.49761
SKPD23_84	34 *	25,459	15.3557
SKPD23_85	38 **	45,613	21.573

*, Pd-positive; **, Pd-inconclusive; ***, Pd not detected.

**Table 3:** Four-gamete test results for individual sample sets of *Pseudogymnoascus destructans*.

	Five Albertan Strains	58 North American Strains	72 Global Strains
Biallelic SNP counts	105	744	216,322
SNP pairs	5460	276,396	23,397,495,681
Four-gamete SNP pairs	0	1207	837,067,329
Ratio of four-gamete SNP pairs over total biallelic SNP pairs	0	0.00437	0.03578

**Table 4:** Colony phenotypes for five strains of Pd isolated from bat wing swabs collected in Alberta in 2024. Data are mean ± standard deviation based on three repeats.

Trait	PdAB3	PdAB6	PdAB8	PdAB9	PdCH2
Colony diameter (mm)	20.33 ± 0.58 ^a^	21.5 ± 0.50 ^a^	21.33 ± 0.58 ^a^	24 ± 0 ^b^	22.33 ± 0.58 ^a^
Pigment diffusion through agar (mm)	50.33 ± 0.58 ^a^	50.67+0.57 ^a^	48.33 ± 0.58 ^a^	39.33 ± 1.15 ^b^	30 ± 0 ^c^
Colony surface exudate production (µL)	15.67 ± 12.09 ^a^	16.33 ± 2.52 ^a^	7.67 ± 0.58 ^b^	10.33+3.51 ^b^	48.67 ± 1.15 ^c^
Exudate pigmentation (visual) *	4 ± 1	5 ± 0	4 ± 0	3.33 ± 1.15	0.33 ± 0.58
Colony surface mycelial pigmentation (visual) **	3 ± 1	3 ± 0	3 ± 0	3 ± 0	2 ± 0

*, scale of 1 to 5, with 1 representing limited exudate production on colony surface and 5 representing large amount of exudate production on colony surface. **, scale of 1 to 5, with 1 representing limited colony surface mycelial pigment production and 5 representing profuse colony mycelial pigmentation production. For each row, the same superscript symbols “a”, “b”, or “c” represent no statistically significant difference among strains while different superscript symbols represent statistically significant differences.

## Data Availability

The genomic sequencing data generated in this study has been deposited in the NCBI Sequence Read Archive (accession numbers SRR35843761 to SRR35843774) under BioProject ID PRJNA1347617.

## Supplementary Materials

The following supporting information can be downloaded at: https://www.mdpi.com/article/10.3390/jof12020154/s1, Table S1: Details of samples and strains collected and analyzed in this study; Table S2: Summary of Pd strain metadata downloaded from the NCBI SRA database; Table S3: Bat species distribution in Canada and their associated mitochondrial cytochrome oxidase subunit I (COI) sequences; Table S4: Coverages and similarities of COI sequences for the 18 Canadian bat species across nine metagenome samples in western Canada; Table S5: Pairwise nucleotide differences among 72 global Pd strains based on whole-genome single nucleotide polymorphisms; Table S6: Copy number estimates of 9620 genes in the Pd genome across all 72 strains along with gene size, average copy number, standard deviation, and coefficient of variation; Table S7: Copy number variated genes (coefficient of variation ≥ 0.5) among North American Pd strains and Gene Ontology annotations provided by RefSeq (NCBI). The Supplementary dataset has been submitted to the Zenodo database under https://doi.org/10.5281/zenodo.18121475.
